# Study on the Mechanism of Nano-SiO_2_ for Improving the Properties of Cement-Based Soil Stabilizer

**DOI:** 10.3390/nano10030405

**Published:** 2020-02-25

**Authors:** Xingchen Zhang, Jianen Gao, Henghui Fan, Xinghua Li, Zhe Gao, Li Xue, Shengli Sun

**Affiliations:** 1 Institute of Soil and Water Conservation, Chinese Academy of Sciences and Ministry of Water Resources, Yangling 712100, China; zxc_cau@163.com (X.Z.);; 2 University of Chinese Academy of Sciences, Beijing 100049, China; 3 Research Center on Soil & Water Conservation, Ministry of Water Resources, Yangling 712100, China; 4 Institute of Soil and Water Conservation, Northwest Agriculture and Forestry University, Yangling 712100, China; 5 College of Water Resources and Architectural Engineering, Northwest Agriculture and Forestry University, Yangling 712100, China

**Keywords:** nano-SiO_2_, soil stabilizer, cement mortars test, hydration reaction, fractal feature

## Abstract

A new nano-soil stabilizer (N-MBER, Nanometer Material Becoming Earth into Rock) material was developed in this research by using the high activity and ultrafine properties of nano-SiO_2_ (NS), which were able to improve the properties of cement-based soil stabilizer and had broad application prospects. The results showed that (1) the strength of N-MBER obeyed a compound function relation with curing period and additive amount of NS. The relationship between strength and curing period obeyed an exponential function when the additive amount was constant. The strength and additive amount were a power function when the curing period was fixed. The compressive strength of N-MBER increased by more than 15% compared with MBER at day 28 of the curing period, and 50% compared with grade 32.5 cement. (2) The pozzolanic catalytic activity of NS significantly increased the amount of calcium silicate hydrate gel (C–S–H) in the N-MBER colloid. NS was also able to make the distribution of the network structure of colloidal space more uniform and improved the fractal dimension of particles by 0.05. The above results provide theoretical data for exploring the mechanism of soil stabilizer strength growth and for promoting the application of solid waste utilization.

## 1. Introduction

With the continuous promotion of global environmental protection, the construction mode of sand mining by digging mountains and rivers to build concrete projects is not only expensive, but also harmful to the local environment. The waste soil and slag from engineering construction could also pollute the environment again, and is difficult to use. In addition, Portland cement and other traditional building materials have higher requirements of sand, stone, and water quality when mixing, which leads to the increase of engineering cost, poor effect of soil stabilization, and aggravation of environmental hazards in the loess plateau and other areas lacking rock and sand [1]. Therefore, there is an urgent need in the field of engineering materials to seek a kind of high-strength, low-energy consumption and environmental protection soil-stabilized material that can make full use of local soil and water resources and has no special water quality requirement.

As a burgeoning kind of environmental protection building material, soil stabilizer has obtained a series of research achievements in water conservancy, road engineering, and other areas in recent years [2,3,4,5,6]. Many scholars have explored the research, development, and application of different soil stabilizers. Fan and Gao et al. [7] analyzed and summarized the types, characteristics, and stabilization mechanisms of the main soil stabilizers, and pointed out that improving the strength of the soil stabilizer is an important direction in future research. Shahnavaz [8] and Zhao et al. [9] studied the effects of different soil stabilizers on soil physical and mechanical properties, but the consolidation mechanism still needs to be further researched. Sharma et al. [10] used a combination of fly ash and slag as stabilizer to modify expansive soil, which showed potential for modification and improvement of soil stabilizers. With the increasing demand for the performance of soil stabilization materials in the fields of engineering application and environmental protection, scholars have made many achievements in exploring the strength improvement of different additives on traditional soil stabilizers [11,12,13,14,15]. Recently, significant achievements had been made in the research on nanoparticle-modified cement, concrete, and other traditional building materials [16,17,18,19,20,21], and positive exploration had also been achieved in the modification of soil stabilizers. Shafiq [22] and Murthy et al. [23] discussed the influence of nano-SiO_2_ on the mechanical properties and durability of concrete. Kong et al. [24] found that nano-SiO_2_ could effectively improve the unconfined compressive strength of loess and improve pore structure. The above explorations have enriched the research on the improvement of the properties of soil stabilizers, but generally speaking, compared with the broad application prospects showed by soil stabilizers as materials for efficient utilization of soil and water resources, the current research status still needs to be deepened. Using new ideas, materials, and technologies to improve the performance of soil stabilizers and further clarify the stabilization mechanism is urgently needed in further research.

Based on the characteristics of nano-SiO_2_, such as high activity and ultrafine properties, this study explored the N-MBER strength enhancement mechanism and hydration process by designing typical tests and physicochemical analysis methods, so as to provide scientific support for the improvement of the engineering performance of soil stabilizers and for the application in the field of environmental protection constructions.

## 2. Materials and Methods

### 2.1. Soil Stabilizer

The soil stabilizer used in this research was MBER, developed by Jianen Gao [1]. MBER is a kind of powdery, environmentally friendly, inorganic cementitious material, which is composed of cement clinker, fly ash, gypsum, active agent, and slag, and is fully ground using a ball mill and then passed through a sieve with a 0.075 mm aperture. This sieve was selected to ensure that the particle size of the mixture was less than 0.075 mm. The cement clinker in the raw materials came from QinLong cement plant in Xi’an, China. The fly ash was first grade fly ash in the power plant, and the gypsum was laboratory grade raw gypsum. The contents of ingredients in MBER and the SO_3_ content in cement clinker are shown in Table 1.

### 2.2. Nano-SiO_2_

The nano-SiO_2_ used in this study, which is commercially available, was a nanomaterial with high purity and activity, and came from the manufacturing industry in Xi’an, China. The average size of the nano-SiO_2_ (NS) particles was about 30 nm, and the basic properties are shown in Table 2. The reasons for choosing NS with particle size of 30nm mainly included: (1) It could fully fill the internal pores of the material. (2) It could make the hydration reaction more sufficient. (3) NS with particle size of 30nm is easy to obtain. 

### 2.3. Sample Preparation

**Figure 1:**
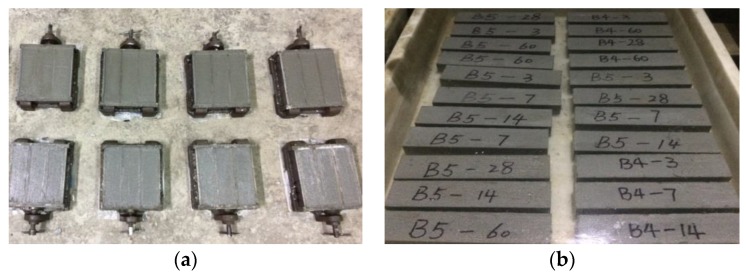
Sample preparation. (**a**) the test blocks in forming; (**b**) the test blocks in curing.

The content of nano-SiO_2_ selected was 1%, 1.5%, 2%, 2.5%, and 3% of the total weight of MBER. Untreated MBER and grade 32.5 cement were used as reference groups. At the beginning of the experiment, tap water was added to the stirring pot with a 0.5 water–cement ratio, and then hydrophilic nano-SiO_2_ was dissolved in water for dispersion and stirring; lastly, quantitative MBER and ISO (International Organization for Standardization) standard sand were added into the pot. The above nanosilica-MBER mortar (N-MBER) was mixed and prepared by a J-55 type planetary mortar mixer. A ZS-15 type cement sand vibrator was used to shape the mixed samples. Figure 1a shows the test blocks after forming. The formed test blocks were put into a fog chamber for curing for 24 h and then demolded. To reduce the error, each of the three test blocks in the same mold were marked as different curing periods. The marked test blocks were put into a constant temperature tank at 20 °C for maintenance, and the water surface was ensured to be 5 mm higher than the test blocks.

### 2.4. Test Procedures

To obtain the mechanical properties of N-MBER, the samples of each period were first tested on the TYE-6A cement mortars bending tester after the curing period, with a loading rate of 50 ± 10 N/s. The broken samples were immediately tested by a TYE-300 pressure testing machine for compressive strength after the breaking strength tests. The samples were uniformly loaded at a rate of 2400 ± 200 N/s until destroyed. To examine the quality of the samples and prevent possible errors, the breaking strength of the specific samples was repeatedly tested three times, and the compressive strength was repeatedly tested six times. The average value of the repeated samples was used in the data analysis. After the strength tests, the fractured samples were carefully collected and sealed. The microstructure and chemical composition of the samples at days 3 and 28 of the curing period were analyzed by S-4800 field emission scanning electron microscopy (SEM) and an energy spectrum analyzer (EDS). All of the tests were conducted in Yangling, ShaanXi Province in China.

## 3. Results and Discussions

### 3.1. Effects of NS on Mechanical Properties of N-MBER

**Figure 2:**
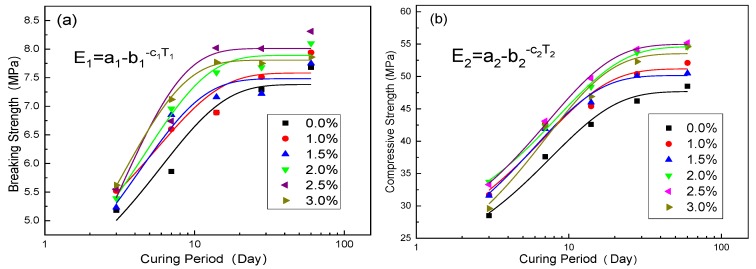
Effects of nano-SiO_2_ (NS) on N-MBER (Nanometer Material Becoming Earth into Rock) strength under different NS content and curing period. (**a**) Breaking strength; (**b**) compressive strength.

Figure 2 shows the breaking strength curve and compressive strength curve of N-MBER with different NS contents under different curing days.

As shown in Figure 2a, a very low NS content can effectively improve the breaking strength of N-MBER at each curing period, and the breaking strength increased significantly at the curing period from 3 days to 14 days; then, its growth rate became gradually stable, and increased slowly at the later stage of curing. The breaking strength of N-MBER was able to reach about 7~9 MPa when the addition amount of NS was 0~3%. Figure 2b shows the effect of NS on the compressive strength of N-MBER. It shows that NS was also able to significantly improve the compressive strength of N-MBER in the early stage of curing. The compressive strength of N-MBER increased by 60~80% from 3 days to 28 days, and then the strength growth rate gradually decreased to about 5% at the curing period from 28 days to 60 days. The statistical analysis function of Origin software was used for variance analysis and F test, and the R square value, residual R-CS, and P value of the correction of determination coefficient were obtained, as shown in Table 3; the results were significant at the level of 0.05. The change trend of compressive strength of N-MBER was basically the same as that of the breaking strength, which showed a positive correlation. The common trend was that the strength increased greatly and rapidly in the early stage, then gradually decreased and became stable in the later stage. Equations (1) and (2), respectively, show the functional relationship between the breaking strength and compressive strength with the curing period of N-MBER.

(1) $$E_{1}=a_{1}-b_{1}^{-c_{1}T_{1}},\;T_{1}\geq 3$$

(2) $$E_{2}=a_{2}-b_{2}^{-c_{2}T_{2}},\;T_{2}\geq 3$$

where $E_{1}$ and $E_{2}$ are the breaking strength and compressive strength of the test blocks, measured in MPa; $T_{1}$ and $T_{2}$ represent the curing period, measured in days; and $a_{1}$, $b_{1}$, $c_{1}$, $a_{2}$, $b_{2}$, and $c_{2}$ are both parameters.

**Figure 3:**
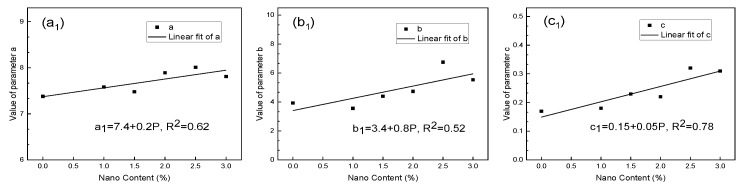
Relationships between breaking strength parameters value and nano content. (**a_1_**) Correlation between parameter a_1_ and NS content; (**b_1_**) Correlation between parameter b_1_ and NS content; (**c_1_**) Correlation between parameter c_1_ and NS content.

**Figure 4:**
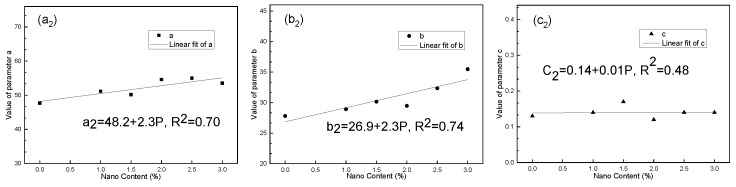
Relationships between compressive strength parameters value and nano content. (**a_2_**) Correlation between parameter a_2_ and NS content; (**b_2_**) Correlation between parameter b_2_ and NS content; (**c_2_**) Correlation between parameter c_2_ and NS content.

The correlation was established between the parameters and the NS content, respectively, and the results are shown in Figure 3 and Figure 4.

By substituting the correlation between parameters and P into Equations (1) and (2), respectively, it could be known:

(3) $$E_{1}=7.4+0.2P-{\left(3.4+0.8P\right)}^{-\left(0.15+0.05P\right)\times T_{1}}$$

(4) $$E_{2}=48.2+2.3P-{\left(26.9+2.3P\right)}^{-\left(0.14+0.01P\right)\times T_{2}}$$

where $E_{1}$ and $E_{2}$ are the breaking strength and compressive strength of the test blocks, measured in MPa; P represents the content of NS, 0%≤P≤3%; $T_{1}$ and $T_{2}$ represent the curing period, measured in days,$T_{1},T_{2}\geq 3$.

As shown in Equations (3) and (4), the breaking strength and compressive strength of N-MBER followed composite function relationships with the NS content and curing period, respectively. When *P* = 0%, N-MBER can be considered as ordinary MBER. Under this condition, the relationship between the strength and the curing period was a kind of exponential function. This indicates that after adding NS, the function expression of the strength growth law was more complex and the reaction inside the material was more sufficient. 

In order to quantitatively analyze the variation law of the strength with curing period under each NS content, assuming *E*_1_ and *E*_2_ were continuously differentiable, Equations (3) and (4) were used to obtain partial derivatives of *T*_1_ and *T*_2_.

The first derivative curve increases monotonically and the second derivative curve decreases monotonically. The results show that the breaking and compressive strengths of N-MBER increased gradually with the growth of curing duration, and the increasing rate of strength tended to become stable gradually with the increase of curing period. E_1_ and E_2_ had maximums of $E_{\max_{1}}$ and $E_{\max_{2}}$. It can be determined from Equations (1) and (2) that $E_{\max_{1}}=a_{1}$ and $E_{\max_{2}}=a_{2}$ were the maximum values calculated by the function models of N-MBER breaking strength and compressive strength at each NS content. The calculated results of the function model show that the N-MBER strength reached the maximum value when the NS content was 2.5%. The maximum breaking strength was 8.01 MPa, and the maximum compressive strength was 54.98 MPa.

### 3.2. Strength of Different Cement-based Soil Stabilizers

This research compared the strength of N-MBER (2.5% NS content), MBER, and 32.5 grade cement. Table 4 shows the cement mortar test results, with compressive strength as the index. It can be seen from Table 4 that the compressive strength of N-MBER at each curing period was the highest under 2.5% NS content, which increased by 14~17% compared with MBER, and by 56~86% compared with grade 32.5 cement.

**Figure 5:**
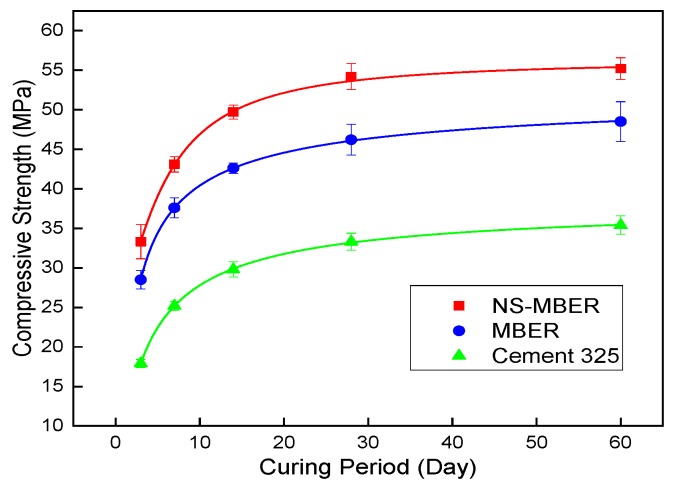
Intensity diagram of NS-MBER, MBER and cement.

The visual fitting tool of Origin software was used to conduct a regression analysis on the changes of compressive strength of the three materials with curing period. As shown in Figure 5, the uniaxial compressive strength variation of cement-based materials can be predicted by the logistic growth curve [25,26]. The model equation for the change of compressive strength of the three cement-based soil stabilizer materials with curing period is shown in Equation (5).

(5) $$E_{c}=\mu -\frac{\mu -\lambda }{1+{\left(\frac{1}{n}T_{c}\right)}^{m}}$$

where *E_c_* is the compressive strength of the material, measured in MPa; *T_c_* represents the curing period, measured in days, and *T_C_* ≥ 3; and λ, μ, *m*, and n are the model parameters.

The degree of fitting analysis provided by Origin software was used to calculate the determination coefficient R^2^ and residual sum of squares (RCS). The closer the R^2^ to 1 and the closer the RCS to 0, the better the fitting effect of the equation. It can be seen from Table 5 that the model can accurately reflect the trend of compressive strength S_C_ of three cement-based soil stabilizer materials with curing period *T_C_*.

### 3.3. Effect of NS on the Hydration Process of Soil Stabilizers

The samples obtained from the cement mortar test were analyzed by S-4800 field emission scanning electron microscopy (SEM) and energy spectrum (EDS) to explain the changes in the microstructure and chemical composition of the samples, and to explore the influence mechanism of NS on the hydration process of N-MBER.

**Figure 6:**
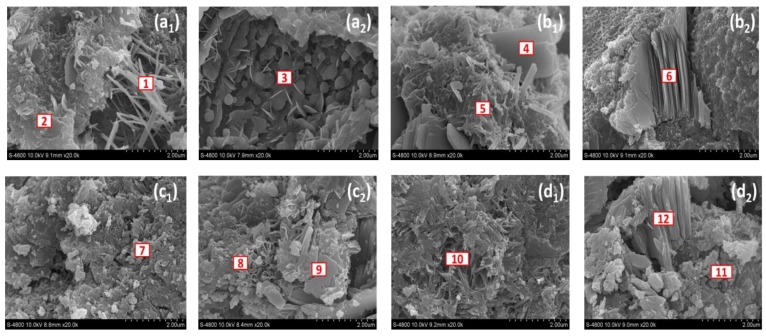
Microstructures of MBER and N-MBER in amplification factor of 20,000. (**a_1_**,**a_2_**) MBER after 3 days; (**b_1_**,**b_2_**) MBER after 28 days; (**c_1_**,**c_2_**) N-MBER after 3 days; (**d_1_**,**d_2_**) N-MBER after 28 days.

Figure 6 shows the SEM images of MBER and N-MBER with 2.5% NS content after 3 days and 28 days of curing, which are at magnification factors of 20,000. As shown in Figure 6(a_1_,a_2_) and Figure 6(c_1_,c_2_), the early curing period SEM images of MBER and N-MBER reveal that NS addition produced an obvious filled effect in pores between particles, which caused a decrease in porosity and an increase in density of the network structure at the phase interface. At the same time, NS caused variations in the hydration reaction inside the samples and changed the type and amount of hydration products.

Figure 6(a_1_,a_2_) and Figure 6(c_1_,c_2_) show the SEM images of MBER and N-MBER during the hydration reaction in 3 days. It can be seen from Figure 6a that there were fibrous or acicular calcium silicate hydrate gel (C–S–H) in the high magnification of MBER at the early period; calcium hydroxide crystals (CH) with large grain size and stratiform or lamellar distribution can also be seen. Figure 6c shows that the addition of NS effectively refined the CH crystals in the hydration products, resulting in a large number of continuous three-dimensional networks of C–S–H gel. With the increase in curing period, the C–S–H can be seen in both Figure 6(b_1_,b_2_) and Figure 6(d_1_,d_2_), while in N-MBER hydration products, the crystal shapes and quantities of C–S–H were more abundant and evenly distributed. At the same time, the amount of CH was significantly reduced, and hexagonal prismatic ettringite (AFt) interlacing was visible. The energy spectrum analysis results of the hydration reaction are shown in Table 6.

The reaction types of volcanic ash mainly included tricalcium silicate (C_3_S), dicalcium silicate (C_2_S), tricalcium aluminate (C_3_A), and tricalcium ferro aluminate (C_4_AF). Calcium silicate hydrate gel (C–S–H), ettringite (AFt), calcium hydroxide (CH), calcium carbonate (CaCO_3_), and other hydration products were generated by the above reactions, and a certain amount of heat was released. The reaction equations are shown in Equations (6) to (9).

3CaO·SiO_2_ + *m*H_2_O = *x*CaO·SiO_2_·yH_2_O + (3−*x*)Ca(OH)_2_ (6)

2CaO·SiO_2_ + *n*H_2_O = *x*CaO·SiO_2_·yH_2_O + (2−*x*)Ca(OH)_2_ (7)

3CaO·Al_2_O_3_ + 6H_2_O = 3CaO·Al_2_O_3_·6H_2_O (8)

4CaO·Al_2_O_3_·Fe_2_O_3_ + 7H_2_O = 3CaO·Al_2_O_3_·6H_2_O + CaO·Fe_2_O_3_·H_2_O (9)

Due to its high activity and ultra-fine properties, the added NS had the effect of inducing hydration and strong volcanic ash activity, which could have caused a secondary hydration reaction with the early hydration products, such as tricalcium silicate C_3_S of N-MBER, and accelerated the hydration process. Furthermore, NS rapidly reacted with CH to generate a large number of C–S–H gels of different shapes, thus improving the interface structure and significantly increasing the strength and performance of N-MBER in each curing period. The reaction equation is shown in Equation (10).

Ca(OH)_2_+SiO_2_+*m*H_2_O = *x*CaO·SiO_2_·yH_2_O (10)

### 3.4. Particle Fractal Characteristics of Stabilized Loess

The microstructure complexity of N-MBER-stabilized loess was reflected by the irregularity, nonlinearity, and uncertainty of its macroscopic properties. Since nano-stabilized soil (N-SS) has typical self-similar characteristics in particle size, pore size, and interface structure, the fractal geometry created by Mandelbrot [27] was adopted for quantitative description, and the fractal dimension value was used as a quantitative parameter, which could serve as a bridge between the microstructure and macroscopic properties of the material. The basic form of the topological dimension is shown in Equation (11).

(11) $$d=\frac{\ln N\left(\varepsilon \right)}{\ln\frac{1}{\varepsilon }}$$

where d is the topological dimension; ε represents the range of yardstick; and N(ε) is the number of times measured by the yardstick, that is, the number of boxes.

To extend the topological dimension to the fractional dimension, it needs to break through the limitation that d is an integer and take the limit of the above Equation (11). The fractal dimension *D* of the box is shown in Equation (12).

(12) $$D=\underset{\varepsilon \to 0}{\lim}\frac{\ln N\left(\varepsilon \right)}{\ln\frac{1}{\varepsilon }}$$

Based on the above theories, the calculation model of fractal dimension D of soil particle distribution proposed by Tyler [28] and Yang peilin [29] was used in this study to analyze the particle fractal characteristics of N-MBER-stabilized soil and MBER-stabilized soil at 90 days of curing. The basic form of the model is shown in Equation (13).

(13) $$\frac{M(\delta <{\bar{d}}_{i})}{M_{0}}={\left(\frac{{\bar{d}}_{i}}{{\bar{d}}_{\max}}\right)}^{3-D}$$

Taking the logarithm of both sides of Equation (13), it could be known that:

(14) $$\log\left[\frac{M\left(\delta <{\bar{d}}_{i}\right)}{M_{0}}\right]=\left(3-D\right)\log\left(\frac{{\bar{d}}_{i}}{{\bar{d}}_{\max}}\right)$$

where ${\bar{d}}_{i}$ is the average size of $d_{i}$ and $d_{i+1}$ of two sieve, measured in mm, ($d_{i}>d_{i+1}$, i=1,2,3…); ${\bar{d}}_{\max}$ represents the maximum diameter of the stabilized soil particle, measured in mm; $M\left(\delta <{\bar{d}}_{i}\right)$ is the volume fraction with a particle size smaller than ${\bar{d}}_{i}$; and $M_{0}$ is the total volume of the stabilized soil particles.

It was assumed that the particle density of the stabilized soil was uniform, so the volume fraction was equal to the mass fraction. Table 7 shows the particle size distribution of the two stabilized soil materials.

**Figure 7:**
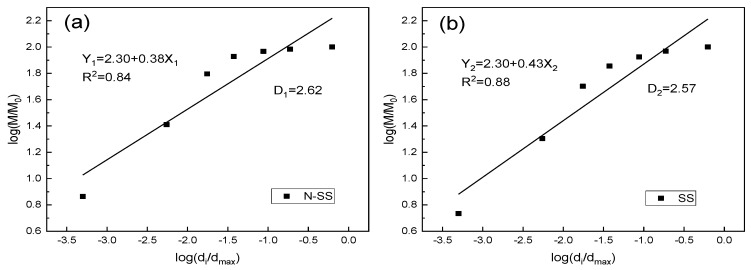
The diagram of $\log\left[M\left(\delta <{\bar{d}}_{i}\right)/M_{0}\right]$−$\log\left({\bar{d}}_{i}/{\bar{d}}_{\max}\right)$. (**a**) N-SS; (**b**) SS.

Figure 7 shows a log–log curve with $\log\left(\frac{{\bar{d}}_{i}}{{\bar{d}}_{\max}}\right)$ and $\log\left[\frac{M\left(\delta <{\bar{d}}_{i}\right)}{M_{0}}\right]$ as horizontal and vertical coordinates. As shown in Figure 7, the particle size distribution data of N-SS and stabilized soil (SS) were fitted into a straight line by linear regression, and the slope (3-D) was calculated to obtain the fractal dimension *D* value of stabilized soil particles.

As is obvious from Figure 7, the fractal dimension of N-SS is 2.62, and that of SS is 2.57. N-SS is obviously larger than SS from the perspective of particle fractal characteristics. Because the fractal dimension of the soil was a parameter reflecting the geometric shape of the soil structure, the larger the fractal dimension was, the smaller the particle size [30]. This indicates that the stabilized soil particles could be effectively refined in terms of geometry, and could be significantly improved at the transition form of the material interface after adding NS. The above result is basically consistent with that by scanning electron microscopy and energy spectrum analysis, which verifies the effects of NS on enhancing the mechanical strength and improving the performance of N-MBER from the perspective of fractal characteristics of stabilized soil particles.

The average particle size of NS selected in this study was 30nm, and the effects of NS particles with other particle sizes on the stabilization effect of cement-based soil stabilizer is still unclear. Since the particle size of NS has certain influence on its properties, such as volcanic activity, fluidity, water adsorption ability, and pore filling properties, the effect of NS particle size on the stabilization effect will be an important research direction in the future.

## 4. Conclusions

The following conclusions were obtained through this research: (1) A composite function model of N-MBER breaking strength and compressive strength under different NS contents and curing periods was proposed. (2) It was found that the strength of N-MBER increased by more than 15% when the content of NS was 2.5%, and the strength increased by 50% compared with that of grade 32.5 cement. (3) The stabilization mechanism of N-MBER was expounded based on microstructure analysis and fractal theory. The results could provide scientific support for further research and application of soil stabilizers.

Soil stabilizers have been successfully applied in the Loess Plateau for more than 10 years, and have obtained a large amount of positive results [31,32,33,34,35], which has brought great convenience to local production and people’s life. The N-MBER proposed on the basis of the above studies could not only significantly improve the strength and performance of the traditional soil stabilizer, but also avoid the mining and transportation of sand and stone, which has advantageous effects on the sustainable development of the local ecological environment. Although this research on nano-soil stabilizers achieved positive results, there were still deficiencies from the following perspectives: (1) In this study, the influence factors of N-MBER on the strength was analyzed from the aspects of NS content and curing period, while other factors, such as moisture content, water quality, and temperature, still need to be further explored. (2) The research on constitutive relation of N-SS is not mature enough, and the physical mechanism of stabilized soil materials needs to be explained. (3) N-MBER has good suitability in Loess regions, but its suitability for other soils is still a subject to be studied. The above deficiencies can provide ideas for future research. 

## Figures and Tables

**Table 1:** Soil stabilizer ingredients and SO_3_ content in the test.

Ingredients	Cement Clinker	Fly Ash	Gypsum	Active Agent	Soil Stabilizer
Content %	85	11	3	1	100
SO_3_ Content	2.01	0.26	1.40	0	3.67

**Table 2:** Basic technical index of nano additive.

Physical Properties	Nano-SiO_2_
Purity (%)	99.9%
APS (nm)	30
SSA (m^2^/g)	600
Colour	white
Morphology	spherical
Bulk Density (g/cm^3^)	0.08
True Density (g/cm^3^)	2.2~2.6

**Table 3:** Statistical analysis results of strength curve fitting.

Statistics		Nano Content %					
		0.0	1.0	1.5	2.0	2.5	3.0
Breaking Strength	Adj.R-Square	0.929	0.888	0.921	0.970	0.900	0.996
	Reduced Chi-Sqr	0.077	0.096	0.072	0.034	0.136	0.003
	P > F	0.005	0.011	0.006	0.001	0.009	0.0001
Compressive Strength	Adj.R-Square	0.977	0.928	0.975	0.995	0.996	0.955
	Reduced Chi-Sqr	1.464	4.721	1.516	0.373	0.329	4.398
	P > F	0.012	0.036	0.013	0.002	0.002	0.022

**Table 4:** Compressive strength of different soil stabilizer.

Materials	Compressive Strength (MPa)				
	3 d	7 d	14 d	28 d	60 d
NS-MBER	33.30	43.10	49.70	54.20	55.20
MBER	28.50	37.60	42.60	46.20	48.50
Cement 325	17.90	25.20	29.80	33.30	35.40

**Table 5:** Calculation results and fitting degree of model parameters.

Materials	λ	μ	*n*	*m*	Statistics	
					Adj.R-Square	Reduced C-S
NS-MBER	25.25	56.12	5.80	1.58	0.97	0.32
MBER	−39.64	52.03	0.69	0.72	0.99	0.01
Cement325	−1.80	38.02	3.07	0.90	0.98	0.02

**Table 6:** Energy spectrum analysis results of MBER and N-MBER.

Point	Metering Mode	C	O	Na	Mg	Al	Si	S	K	Ca	Fe	Mineral Type
1	Wt%	5.00	43.62	0.74	0.77	3.74	10.04	0.60	0.94	31.53	3.02	C-S-H
	At%	9.08	59.44	0.70	0.69	3.02	7.80	0.41	0.53	17.15	1.18	
2	Wt%	5.00	43.72	0.85	0.87	3.84	9.85	0.58	1.08	31.54	2.67	CH
	At%	9.06	59.46	0.81	0.78	3.09	7.63	0.40	0.60	17.13	1.04	
3	Wt%	4.78	43.73	0.83	0.76	3.76	9.83	0.63	1.02	31.97	2.69	CH
	At%	8.68	59.69	0.79	0.69	3.04	7.64	0.43	0.57	17.42	1.05	
4	Wt%	7.85	45.85	1.06	0.52	4.10	10.23	0.69	1.12	27.68	0.92	AFt
	At%	13.44	58.97	0.94	0.44	3.13	7.50	0.44	0.59	14.21	0.34	
5	Wt%	7.77	45.76	0.95	0.53	4.21	10.3	0.72	1.12	27.58	1.07	C-S-H
	At%	13.34	58.96	0.85	0.45	3.22	7.56	0.46	0.59	14.19	0.39	
6	Wt%	7.92	45.87	1.07	0.56	4.18	10.33	0.50	0.90	27.77	0.89	AFt
	At%	13.56	58.91	0.95	0.48	3.18	7.56	0.32	0.48	14.23	0.33	
7	Wt%	5.29	46.43	0.82	0.46	1.53	7.74	0.45	0.84	35.31	1.14	C-S-H
	At%	9.43	62.20	0.76	0.40	1.22	5.91	0.30	0.46	18.88	0.44	
8	Wt%	3.91	38.53	1.58	0.48	2.59	16.38	0.72	1.82	32.78	1.20	C-S-H
	At%	7.39	54.61	1.56	0.45	2.18	13.22	0.51	1.06	18.54	0.49	
9	Wt%	3.72	42.95	0.98	0.07	1.53	16.70	0.23	1.52	32.30	0.00	CH
	At%	6.83	59.09	0.94	0.06	1.24	13.09	0.16	0.86	17.74	0.00	
10	Wt%	5.22	41.74	0.71	1.87	4.33	11.11	0.59	0.75	30.05	3.62	C-S-H
	At%	9.53	57.22	0.68	1.69	3.52	8.67	0.4	0.42	16.45	1.42	
11	Wt%	5.17	41.99	0.76	1.82	4.30	10.73	0.62	0.80	30.26	3.55	C-S-H
	At%	9.43	57.52	0.73	1.64	3.50	8.38	0.42	0.45	16.55	1.39	
12	Wt%	5.08	41.68	0.78	1.99	4.45	10.98	0.51	0.68	30.17	3.68	AFt
	At%	9.29	57.24	0.75	1.80	3.63	8.59	0.35	0.38	16.54	1.45	

**Table 7:** Particle size distribution of nano-stabilized soil (N-SS) and stabilized soil (SS).

Materials	The Ratio of Particle Volume to Total Volume in Different Particle Sizes						
	<0.002	0.002~0.02	0.02~0.05	0.05~0.1	0.1~0.25	0.25~0.5	0.5~2
N-SS	8.88	17.58	32.30	18.74	7.432	5.92	9.14
SS	7.87	16.16	30.67	20.62	11.11	8.033	5.53
